# The Relationship Between Maternal Serum Afamin Levels and Intrahepatic Cholestasis of Pregnancy and Neonatal Outcomes

**DOI:** 10.3390/jcm15093241

**Published:** 2026-04-24

**Authors:** Kubilay Çanga, Bengisu Elüstü, İbrahim Buğra Bahadır, Ümran Özcan, Seyit Ahmet Erol, Şevki Çelen

**Affiliations:** Division of Perinatology, Department of Obstetrics and Gynecology, Etlik City Hospital, Ministry of Health, Ankara 06170, Türkiye; bengisucakr@gmail.com (B.E.); ibbahadir@hotmail.com (İ.B.B.); ozcanumran38@gmail.com (Ü.Ö.); gyn.aerol@gmail.com (S.A.E.); sevkicelen@yahoo.com (Ş.Ç.)

**Keywords:** intrahepatic cholestasis of pregnancy, afamin, bile acids

## Abstract

**Objective**: This study aimed to evaluate maternal serum afamin levels in women with intrahepatic cholestasis of pregnancy (ICP), examine their relationship with fasting bile acid concentrations, and assess their association with perinatal outcomes. **Methods**: This prospective case-–control study included 80 singleton pregnancies followed at a tertiary perinatology center between October 2025 and March 2026. Forty women with ICP, defined by pruritus and fasting bile acids > 10 μmol/L, were compared with 40 healthy pregnant controls. Women with ICP were further stratified according to fasting bile acid levels as <40 and ≥40 μmol/L. Maternal serum afamin concentrations were measured using a commercially available enzyme-linked immunosorbent assay (ELISA) kit. Maternal characteristics, liver biochemistry, fetal biometric and Doppler parameters as well as obstetric and neonatal outcomes were compared. Receiver operating characteristic (ROC) curve analysis was performed to evaluate the diagnostic performance of afamin for ICP, and logistic regression analysis was used to assess its association with ICP. **Results**: Baseline maternal characteristics were comparable between groups. Maternal serum afamin levels were significantly higher in the ICP group than in controls (6.18 ± 4.24 vs. 3.98 ± 1.95 ng/mL, *p* = 0.004). Afamin correlated positively with fasting bile acids (r = 0.372, *p* = 0.018), but not with transaminases, gestational age at delivery, birth weight, or neonatal outcomes. In logistic regression, afamin was independently associated with ICP (adjusted odds ratio [aOR] 1.260; 95% confidence interval [CI] 1.059–1.500; *p* = 0.009). ROC analysis showed poor discrimination for ICP (area under the curve [AUC] 0.634, 95% CI 0.51–0.76, *p* = 0.039), whereas afamin did not discriminate between subgroups defined by fasting bile acid levels (<40 vs. ≥40 μmol/L). The optimal cut-off value of 4.93 ng/mL predicted ICP with 55% sensitivity, 67.5% specificity, a positive likelihood ratio of 1.69, and a negative likelihood ratio of 0.67. **Conclusions**: Maternal serum afamin levels are elevated in ICP and show a modest association with fasting bile acid burden. Its discriminatory performance is limited, and it does not reliably distinguish patients defined by a ≥40 μmol/L threshold. These findings suggest that afamin reflects the maternal response to cholestasis rather than disease severity and may serve as a complementary biomarker.

## 1. Introduction

Intrahepatic cholestasis of pregnancy is a pregnancy-specific liver disorder that typically emerges in the late second or third trimester. It is defined by maternal pruritus together with elevated serum bile acid levels [1]. In clinical practice, management is mainly guided by bile acid concentrations, symptom severity, and gestational age at diagnosis [1]. Maternal symptoms and laboratory abnormalities usually improve after delivery. Despite this, ICP remains clinically important because it is associated with adverse perinatal outcomes, including spontaneous or iatrogenic preterm birth, meconium-stained amniotic fluid, neonatal respiratory complications, and stillbirth [2,3,4]. A key difficulty in managing ICP is the mismatch between maternal and fetal status: while the mother may appear relatively well, the fetus is still exposed to a potentially harmful intrauterine environment.

Bile acids are central to the evaluation and clinical management of ICP. However, they do not provide sufficient information on their own. These compounds do not reflect the biological heterogeneity of the disease, nor do they fully reveal the mother’s overall response to cholestatic stress. Current evidence supports a multifactorial pathogenesis. Genetic susceptibility appears to contribute to disease severity in a subset of women, including those carrying variants in hepatobiliary transport genes such as ABCB4 [5]. Bile acids may also play a direct pathophysiologic role. Cholestasis can impair placental transport mechanisms and reduce fetal-to-maternal bile acid clearance [6]. Bile acids have also been shown to cause dose-dependent vasoconstriction in placental chorionic veins, which may help explain acute fetal compromise even when classical features of chronic placental insufficiency are absent [7]. In parallel, cholestatic stress has been linked to placental oxidative injury, apoptosis, inflammatory activation, endoplasmic reticulum stress, and altered angiogenic pathways in both animal and human studies [8,9,10]. Recent human studies indicate that higher maternal bile acid levels may be linked to impaired fetal cardiac function. This supports the view that ICP is better understood as a maternal–placental–fetal stress condition than as an isolated maternal liver disorder [11].

Afamin belongs to the albumin gene family and functions as a vitamin E-binding glycoprotein. This makes it biologically relevant in conditions marked by oxidative imbalance and altered extracellular antioxidant transport [12]. It is produced mainly in the liver, rises progressively during normal pregnancy, and has not been detected in placental tissue in uncomplicated gestations [13]. These features suggest that afamin may be a marker of the maternal hepatic and oxidative response to cholestasis, rather than being produced by the placenta. In this context, it could offer insight into a disorder influenced by the interplay of maternal liver dysfunction, metabolic stress, and placental vulnerability.

Outside of pregnancy-specific liver disease, higher circulating afamin levels have been associated with metabolic syndrome, insulin resistance, polycystic ovary syndrome, and incident type 2 diabetes. These conditions are characterized by metabolic strain and a disrupted redox balance [14,15,16]. This pattern suggests that afamin reflects underlying oxidative and metabolic stress rather than a single disease process. This is especially important in ICP, where the primary means of assessing patients is measuring bile acid levels. But the results of treatment are probably affected by a wider range of responses in the liver, blood vessels, the body’s reaction to infection, and the placenta.

In obstetric research, first-trimester afamin levels have been independently associated with the subsequent development of pre-eclampsia and gestational diabetes. This suggests that afamin could play a role in identifying pregnancies at increased risk of oxidative and metabolic vulnerability before clinical disease becomes apparent [17]. Despite this biological rationale, the relationship between maternal serum afamin and ICP remains poorly characterized. This is clinically relevant as the current assessment of ICP largely relies on bile acid levels, which may not fully capture the broader oxidative and metabolic disturbances underlying the disease.

This study investigated maternal serum afamin levels in women with ICP and in healthy pregnancies, and explored their relationship with bile acid levels and adverse perinatal outcomes.

## 2. Materials and Methods

This prospective case-–control study was conducted at Ankara Etlik City Hospital, a tertiary maternal–fetal medicine center, between October 2025 and March 2026. Eligible participants were consecutively recruited from both the perinatology outpatient clinic and the inpatient service during the study period. Eighty women were included, with 40 in the ICP group and 40 healthy controls. The study was approved by the Scientific Research Evaluation and Ethics Committee of Ankara Etlik City Hospital (30 September 2025; AEŞH-BADEK2-2025-334). All participants provided written informed consent, and the study followed the principles of the Declaration of Helsinki.

The case group included women with ICP, while the control group comprised healthy pregnant women followed in the same unit. According to the 2020 Society for Maternal-Fetal Medicine (SMFM) guideline, a fasting serum bile acid level of ≥10 μmol/L is accepted as a practical threshold for diagnosing ICP in pregnancy [18]. In this study, ICP was diagnosed in women with new-onset pruritus without a primary skin rash and fasting total bile acid levels > 10 μmol/L, after exclusion of alternative hepatobiliary and systemic causes. Based on the same guideline and prior outcome-based literature, women with ICP were further stratified according to fasting bile acid levels as <40 and ≥40 μmol/L, since levels ≥ 40 μmol/L are associated with an increased risk of adverse perinatal outcomes [4,18]. Healthy controls were selected from singleton pregnancies without pruritus, cholestasis, or maternal or fetal conditions likely to affect hepatobiliary, metabolic, inflammatory, or placental function. Blood sampling was performed at approximately 32 weeks’ gestation.

Inclusion criteria were singleton pregnancy, maternal age ≥ 18 years, availability of fasting bile acid measurement, and availability of serum samples for afamin analysis. Exclusion criteria were multifetal gestation, major fetal anomaly or aneuploidy, preexisting liver disease, viral hepatitis, obstructive biliary disease or gallstones, chronic inflammatory or autoimmune disease, chronic renal disease, pregestational diabetes, gestational diabetes, preeclampsia or hemolysis, elevated liver enzymes, and low platelets (HELLP) syndrome, and initiation of ursodeoxycholic acid (UDCA) before the baseline blood sample. Women with incomplete clinical, laboratory, ultrasonographic, or outcome data were not included. Consequently, 40 women with ICP were included in the final analysis. In addition, 40 healthy pregnant women, who were consecutively recruited and matched 1:1 for maternal age, gestational age, pregestational body mass index (BMI), and gravidity, were included as the control group.

At the initial evaluation, maternal demographic and obstetric characteristics were recorded. Recorded variables included maternal age, gravidity, parity, nulliparity, BMI, gestational weight gain, gestational age at sampling, and the time from sampling to delivery. Venous blood samples were obtained when women attended the perinatology clinic or were admitted for suspected ICP. All baseline samples were collected before initiation of medical therapy and after an overnight fast of at least 8 h. Routine biochemical assessment included fasting total bile acids, alanine aminotransferase (ALT), aspartate aminotransferase (AST), albumin, prothrombin time (PT), activated partial thromboplastin time (aPTT), international normalized ratio (INR), and fibrinogen. In women with ICP, additional cholestatic markers and follow-up liver tests were also recorded, including gamma-glutamyl transferase (GGT), alkaline phosphatase (ALP), bilirubin fractions, predelivery transaminases, and transaminases measured one week postpartum.

For afamin measurement, venous blood samples were obtained from the antecubital vein. After allowing the samples to clot at room temperature, they were centrifuged at 1000× *g* for 20 min. Serum was separated after centrifugation, aliquoted into sterile microtubes, and stored at −80 °C until analysis, avoiding repeated freeze–thaw cycles. Maternal serum afamin levels were measured using a commercially available ELISA kit (Human AFM ELISA Kit, Cat No: ATO3008; AtoChemBio, Butler, NJ, USA), based on the sandwich immunoassay principle. The assay covered a detection range of 3.13–200 ng/mL, with a sensitivity of 1.48 ng/mL. All samples and reagents were brought to room temperature before analysis. Each sample was analyzed in duplicate wells, and the mean of the two measurements was used for concentration calculation to improve analytical reliability. Optical density was measured at 450 nm using a microplate reader, and concentrations were derived from the standard curve according to the manufacturer’s instructions. Results were reported in ng/mL. The intra-assay and inter-assay coefficients of variation (CVss) were reported by the manufacturer to be <8% and <10%, respectively.

The primary aim was to compare maternal serum afamin levels between women with ICP and healthy controls, and to assess whether afamin was independently associated with ICP. Secondary objectives were to evaluate the relationship between afamin and fasting bile acid concentrations, compare women with fasting bile acid levels < 40 and ≥40 μmol/L, and assess obstetric and neonatal outcomes. Combined adverse perinatal outcome (CAPO) was prespecified as a secondary outcome. It was defined as the presence of at least one of the following: preterm birth, fetal distress, neonatal acidemia (umbilical cord pH < 7.20), neonatal intensive care unit (NICU) admission, transient tachypnea of the newborn (TTN), need for respiratory support (continuous positive airway pressure [CPAP] or mechanical ventilation), phototherapy, or neonatal hypoglycemia.

### 2.1. Sample Size Calculation

Sample size and statistical power were determined using G*Power software (version 3.1.9.7; Universität Düsseldorf, Düsseldorf, Germany). Based on a previously reported effect size of d = 0.78, a minimum total sample size of 72 participants was calculated to achieve 90% statistical power at a two-sided α level of 0.05 [19]. In a 1:1 case-–control design, this corresponded to at least 36 women with ICP and 36 controls. To improve the statistical reliability and increase the precision of effect size estimates, the target sample size was expanded to 80 participants, and the study was therefore designed to include 40 cases and 40 controls.

### 2.2. Statistical Analysis

Data were analyzed using IBM SPSS Statistics for Windows, version 27.0 (IBM Corp., Armonk, NY, USA). Normality was evaluated with visual methods and the Shapiro–Wilk test. Continuous variables were presented as mean ± standard deviation or median (interquartile range), depending on distribution. Group comparisons were performed using the Student *t*-test or the Mann–Whitney U test. Categorical variables were compared with the Pearson chi-square or Fisher’s exact test. Correlation analyses were conducted in line with data distribution. ROC analysis was used to evaluate the predictive performance of afamin for ICP and for ICP with fasting bile acid levels ≥ 40 μmol/L. Area under the ROC curve values with 95% confidence intervals were calculated, and because no predefined clinical threshold exists for afamin, the optimal cut-off value was derived post hoc using the Youden index. Univariable logistic regression was followed by multivariable analysis adjusted for gestational age at sampling, maternal age, pregestational body mass index, and gestational weight gain. Results were reported as odds ratios with 95% confidence intervals. A two-sided *p*-value < 0.05 was considered statistically significant.

## 3. Results

**Figure 1:**
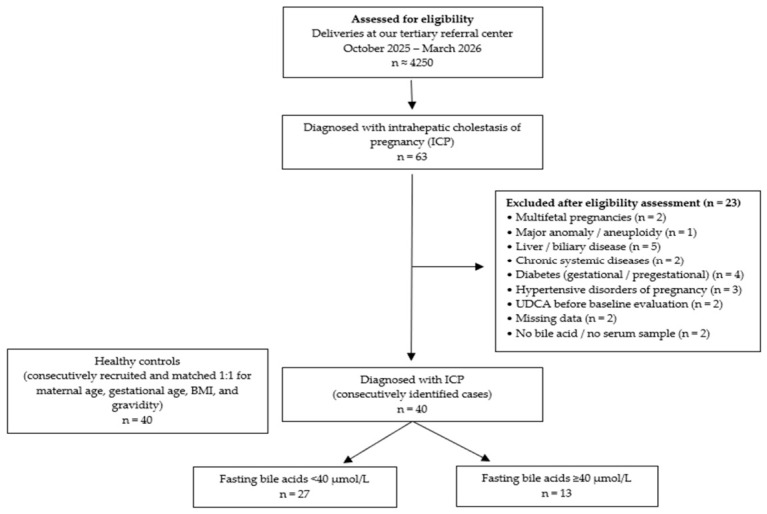
Participant flow diagram of study enrollment, exclusions, and subgroup allocation.

A total of approximately 4250 deliveries at our tertiary referral center between October 2025 and March 2026 were assessed for eligibility. Among these, 63 women were diagnosed with ICP. After applying the predefined exclusion criteria, 23 patients were excluded due to multifetal pregnancy (*n* = 2), major fetal anomaly or aneuploidy (*n* = 1), liver or biliary disease (*n* = 5), chronic systemic disease (*n* = 2), diabetes (gestational or pregestational) (*n* = 4), hypertensive disorders of pregnancy (*n* = 3), use of UDCA prior to baseline evaluation (*n* = 2), missing data (*n* = 2), and lack of bile acid measurement or serum sample (*n* = 2). Consequently, 40 women with ICP were included in the final analysis. In addition, 40 healthy pregnant women, who were consecutively recruited and matched 1:1 for maternal age, gestational age, and gravidity, were included as the control group. The ICP cohort was further stratified according to fasting bile acid levels into <40 µmol/L (*n* = 27) and ≥40 µmol/L (*n* = 13) subgroups. The participant flow and subgroup allocation are summarized in Figure 1.

Eighty pregnant women were included, with 40 in the ICP group and 40 in the control group. Maternal age, gravidity, parity, nulliparity rate, pregestational BMI, gestational weight gain, gestational age at sampling, and gestational age at ultrasound were similar between groups. However, the time from blood sampling to delivery was significantly shorter in the ICP group (3 vs. 6 weeks, *p* = 0.002) (Table 1).

No significant between-group differences were observed in fetal biometric or Doppler parameters. Estimated fetal weight, estimated fetal weight percentile, maximum vertical pocket, umbilical artery pulsatility index (PI), and umbilical artery systolic/diastolic (S/D) ratio were comparable between women with ICP and controls (all *p* > 0.05) (Table 2).

Maternal serum afamin levels were significantly higher in the ICP group than in the control group (6.18 ± 4.24 vs. 3.98 ± 1.95, *p* = 0.004). Fasting bile acid concentrations were also markedly higher in women with ICP (34.93 ± 29.27 vs. 4.49 ± 1.65, *p* < 0.001), together with higher ALT and AST levels (both *p* < 0.001). Albumin levels were lower in the ICP group (35.54 ± 2.73 vs. 37.01 ± 3.16, *p* = 0.029), whereas PT, aPTT, and INR were similar between groups. Fibrinogen levels were significantly higher in women with ICP (596.25 ± 108.33 vs. 428.03 ± 64.61, *p* < 0.001). GGT, ALP, total bilirubin, and direct bilirubin were reported only in the ICP group and therefore were not compared between groups (Table 3).

Regarding obstetric and neonatal outcomes, women with ICP delivered earlier than controls (37 vs. 39 weeks, *p* < 0.001), and preterm delivery was more frequent in the ICP group (25.0% vs. 5.0%, *p* = 0.025). Birth weight was lower in pregnancies complicated by ICP (2858 ± 527 vs. 3305 ± 456 g, *p* < 0.001). Umbilical cord arterial pH was lower in the ICP group (7.33 vs. 7.35, *p* = 0.042). A significant between-group difference was observed for the 5 -min Apgar score (*p* = 0.044), whereas the difference in 1 -min Apgar score was borderline (*p* = 0.050). Antenatal corticosteroid administration was more frequent in the ICP group (35.0% vs. 0%, *p* < 0.001). Cesarean delivery, fetal distress, NICU admission, TTN, CPAP requirement, and phototherapy requirement did not differ significantly between groups. There were no cases of respiratory distress syndrome (RDS), mechanical ventilation, neonatal hypoglycemia, neonatal sepsis, or perinatal mortality in either group. However, the rate of CAPO was significantly higher in the ICP group than in controls (37.5% vs. 12.5%, *p* = 0.010) (Table 4).

Among women with ICP, 27 had fasting bile acid levels < 40 µmol/L and 13 had levels ≥ 40 µmol/L. Afamin levels did not differ significantly between the two subgroups (5.71 ± 4.24 vs. 7.16 ± 4.25, *p* = 0.318). Women with bile acid levels ≥ 40 µmol/L had higher fasting bile acid concentrations (68.40 ± 29.96 vs. 18.81 ± 6.42, *p* < 0.001), higher ALT levels (225 vs. 87, *p* = 0.031), and required higher doses of ursodeoxycholic acid (4 vs. 3 tablets/day, *p* = 0.006). No significant differences were found between the subgroups in gestational age at sampling, AST, GGT, ALP, bilirubin levels, albumin, pre-delivery and postpartum transaminase levels, hepatic ultrasonographic findings, estimated fetal weight percentile, umbilical artery PI, time from sampling to delivery, birth weight, NICU admission, NICU length of stay, or CAPO rates (Table 5).

Correlation analysis showed a positive correlation between maternal serum afamin levels and fasting bile acid concentrations (r = 0.372, *p* = 0.018). No significant correlations were found between afamin levels and maternal age, gestational age at blood sampling, pre-gestational BMI, ALT, AST, total bilirubin, albumin, estimated fetal weight percentile, umbilical artery PI, gestational age at delivery, birth weight, 5 -min Apgar score, or NICU admission (Table 6).

**Figure 2:**
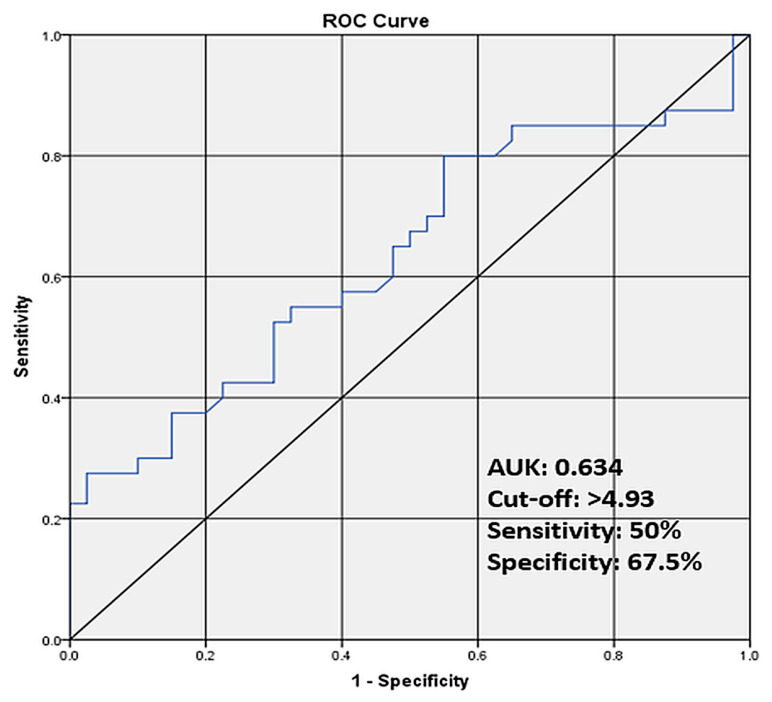
ROC curve of maternal serum afamin levels for the prediction of intrahepatic cholestasis of pregnancy.

ROC analysis showed that maternal serum afamin had an AUC of 0.634 (95% CI, 0.51–0.76; *p* = 0.039) for predicting ICP. Because no predefined clinical threshold exists for afamin, the cut-off value of >4.93 was derived post hoc using the Youden index. Sensitivity was 55.0%, and specificity was 67.5%, with a positive likelihood ratio of 1.69 and a negative likelihood ratio of 0.67 (Table 7, Figure 2). In contrast, afamin was not a significant predictor of severe ICP, defined as fasting bile acid levels ≥ 40 µmol/L (AUC 0.593, 95% CI 0.40–0.78, *p* = 0.348).

In univariable logistic regression, afamin was significantly associated with ICP (odds ratio [OR] 1.254, 95% CI 1.059–1.485, *p* = 0.009). This relationship remained significant after adjustment for gestational age at sampling, maternal age, pregestational BMI, and gestational weight gain (aOR 1.260, 95% CI 1.059–1.500, *p* = 0.009). None of the other variables showed a significant association with ICP (Table 8).

## 4. Discussion

In this prospective case-–control study, maternal serum afamin concentrations were significantly higher in women with ICP than in healthy pregnant controls. Afamin also showed a positive correlation with fasting bile acid levels and remained independently associated with the presence of ICP after adjustment for major maternal covariates. By contrast, its discriminatory performance was modest. Moreover, the observed AUC of 0.634 falls below the commonly accepted threshold of 0.70 for clinically useful discrimination, indicating poor standalone diagnostic utility. It did not reliably distinguish women with fasting bile acid levels < 40 μmol/L from those with levels ≥ 40 μmol/L. Clinically, pregnancies complicated by ICP were characterized by earlier delivery, higher rates of preterm birth, lower birth weight, lower umbilical cord arterial pH, and a higher rate of composite adverse perinatal outcome. Taken together, these findings suggest that afamin may reflect the maternal response to cholestatic stress in ICP, but is unlikely to serve as a strong standalone marker of disease severity or neonatal risk.

Afamin is a liver-derived vitamin E-binding glycoprotein that rises physiologically during pregnancy. In uncomplicated pregnancies, it has not been shown to be expressed in placental tissue [13]. This makes afamin particularly relevant in ICP, because any rise in circulating levels is more likely to reflect the maternal hepatic and oxidative–metabolic response than direct placental production [13]. ICP itself should not be viewed only as maternal itching accompanied by abnormal liver tests. It is increasingly understood as a maternal–placental–fetal stress syndrome in which bile acid accumulation affects placental vascular tone, oxidative balance, and fetal cardiac function [7,8,11]. Experimental and clinical studies show that bile acids can cause placental vasoconstriction. They also promote oxidative injury and apoptosis. In addition, they may impair fetal cardiac function [7,8,11,20]. These findings provide a biologically plausible basis for investigating afamin in ICP.

Our finding of higher afamin levels in ICP is in line with the general literature, even though studies specifically on ICP are limited. In nonpregnant populations, afamin has been linked to liver fat accumulation, insulin resistance, and nonalcoholic fatty liver disease. This supports the idea that afamin reflects hepatometabolic stress rather than a single disease [21,22]. In obstetric studies, higher afamin levels have been consistently reported across several pregnancy complications, including preeclampsia and gestational diabetes mellitus. First-trimester afamin concentrations are significantly elevated in women who subsequently develop preeclampsia [23], while increased afamin levels in early pregnancy have been associated with a higher risk of gestational diabetes [24,25]. In addition, elevated maternal serum afamin concentrations have also been reported in pregnancies complicated by fetal growth restriction, suggesting a broader association with adverse placental and metabolic conditions [26]. Although these conditions differ from one another, they share common features such as maternal dysregulation, oxidative stress, and overall metabolic burden. Our findings also suggest that ICP may form part of a broader biological picture in which afamin levels rise in response to maternal liver and metabolic stress.

One of the key findings of our study was a positive correlation between afamin and fasting bile acids. In contrast, no significant correlation was found between afamin and ALT or AST. In our opinion, this is an important point rather than a minor detail. In ICP, bile acids are the principal biochemical hallmark of disease and the laboratory parameter most consistently linked to fetal risk, whereas transaminases may fluctuate considerably and do not always parallel clinical severity [4,27,28]. At the same time, afamin appears to be more closely related to hepatic lipid accumulation and metabolic dysfunction than to general adiposity alone [21,22]. Our findings therefore support the interpretation that afamin in ICP may be more closely aligned with cholestatic and oxidative–metabolic burden than with hepatocellular injury itself. In that sense, afamin may provide information beyond conventional liver enzymes by capturing a different and potentially more pathophysiologically relevant aspect of the maternal response.

Bile acid concentrations ≥ 40 μmol/L have long been used to denote a higher cholestatic burden and to identify women at greater risk of adverse pregnancy outcomes [4,28,29,30,31]. More recent work has refined this view. Non-fasting sampling may identify high-risk pregnancies more effectively, and the strongest association with stillbirth is seen at bile acid levels ≥ 100 μmol/L [4,28,29,31]. Even so, our cohort was based on fasting pretreatment bile acid measurements. Within that framework, the 40 μmol/L threshold remained a reasonable and literature-supported cut-off for comparing lower and higher biochemical burden [28,29,30,31]. The absence of a significant difference in afamin levels between these subgroups suggests that afamin may reflect the presence of ICP-related maternal cholestatic and oxidative–metabolic stress rather than a simple stepwise increase in bile acid burden. However, the relatively small subgroup size may also have limited our ability to detect such a gradient.

The obstetric and neonatal findings in our study were broadly consistent with the current ICP literature. Women with ICP delivered earlier, had more preterm births, and delivered neonates with lower birth weight. CAPO was also more frequent in the ICP group. Recent systematic reviews, meta-analyses, and severity-based cohort studies have similarly shown increased risks of preterm birth, NICU admission, lower birth weight, meconium-stained amniotic fluid, and other adverse outcomes as bile acid burden rises [4,29,30,31,32]. At the same time, neonatal outcomes in ICP are shaped not only by disease biology but also by clinical management. In our cohort, the earlier timing of delivery, the shorter interval from blood sampling to delivery, and the higher frequency of antenatal corticosteroid use in the ICP group suggest that part of the excess in preterm birth, lower birth weight, and some neonatal outcomes may reflect planned obstetric intervention rather than disease biology alone. This is particularly important because CAPO includes preterm birth, which in ICP may be both spontaneous and iatrogenic. Therefore, attributing all neonatal outcomes solely to afamin or bile acid levels may be misleading.

This study has several strengths. It was designed prospectively, and all samples were collected before treatment. This helped reduce treatment-related bias. Both cases and controls were recruited from the same tertiary center, which improved consistency between groups. Gestational age at sampling was also similar. This is important because afamin levels naturally increase during pregnancy. Several limitations should also be acknowledged. This was a single-center study with a modest sample size. Although pregestational BMI was recorded and included in the multivariable analysis, residual confounding related to adiposity, metabolic status, diet, and other unmeasured maternal characteristics cannot be excluded. In addition, the subgroup with fasting bile acids ≥ 40 μmol/L included only 13 women, so severity-based subgroup analyses were underpowered and should be interpreted cautiously. Afamin was measured only once, so its temporal dynamics could not be assessed. In addition, reliance on fasting bile acid measurements may have led to underestimation of disease burden. Furthermore, some afamin measurements fell below the lower limit of quantification of the assay; although numerical values were obtained through curve extrapolation, these low-level measurements may be associated with reduced analytical accuracy and should therefore be interpreted with caution.

Overall, our findings suggest that maternal serum afamin is elevated in ICP and is more likely to reflect the maternal hepatic and oxidative–metabolic response to cholestasis than to function as a precise marker of disease severity or neonatal prognosis. Rather than competing with bile acids, afamin may capture a complementary biologic dimension of ICP. Larger studies with serial measurements and separate analyses based on fasting, non-fasting, and very high bile acid levels are needed to determine whether afamin can contribute meaningfully to multimarker risk stratification models in ICP.

## 5. Conclusions

Maternal serum afamin levels are elevated in pregnancies complicated by ICP and show a modest association with fasting bile acid burden. Although afamin was independently associated with the presence of ICP, its discriminatory performance was limited, and it did not reliably distinguish women defined by fasting bile acid concentrations ≥40 μmol/L. Given that the observed discriminatory performance did not reach the level generally considered acceptable for clinical usefulness, afamin demonstrates poor standalone diagnostic value. Our findings suggest that afamin reflects the maternal hepatic and oxidative–metabolic response to cholestasis more than disease severity itself. Afamin should therefore be viewed as a complementary biomarker rather than an alternative to bile acid measurement. Larger prospective studies with serial measurements are needed to define its place in multimarker risk assessment for ICP.

## Figures and Tables

**Table 1:** Maternal demographic and clinical characteristics of women with intrahepatic cholestasis of pregnancy and controls.

Variables	ICP (*n* = 40)	Control (*n* = 40)	*p*
Maternal age (years)	29.8 ± 5.2	30.1 ± 5.3	0.832 ^a^
Gravida	2 (2)	2 (1)	0.231 ^b^
Parity	1 (1)	1 (2)	0.175 ^b^
Nulliparity	20 (50%)	13 (32.5%)	0.112 ^c^
Pre-gestational BMI (kg/m^2^)	27.2 ± 4.6	26.9 ± 4.1	0.781 ^a^
Gestational weight gain (kg)	10 (5)	12 (5.5)	0.203 ^b^
GA at blood sampling (weeks)	32 (5)	32 (6)	0.690 ^b^
GA at ultrasound examination (weeks)	32 (5)	32 (6)	0.690 ^b^
Time from blood sampling to delivery (weeks)	3 (4)	6 (5)	0.002 ^b^

Data are expressed as *n* (%), mean ± SD, or median (interquartile range) where appropriate. A *p*-value of <0.05 indicates a significant difference. ^a^: Student *t* test; ^b^: Mann–Whitney U test; ^c^: Pearson chi-square test. Abbreviations: ICP: intrahepatic cholestasis of pregnancy; *n*: number; BMI: body mass index; kg: kilogram; m: meter; GA: gestational age.

**Table 2:** Fetal biometric and Doppler parameters in intrahepatic cholestasis of pregnancy and control groups.

Variables	ICP (*n* = 40)	Control (*n* = 40)	*p*
EFW (gram)	2025 ± 575	2150 ± 706	0.387 ^a^
EFW percentile	45.2 ± 20.9	51.2 ± 19.9	0.194 ^a^
MVP	55 (19)	50 (13)	0.238 ^b^
UA PI	0.96 ± 0.21	0.91 ± 0.20	0.222 ^a^
UA S/D	2.55 ± 0.44	2.63 ± 0.43	0.439 ^a^

Data are expressed as mean ± SD or median (interquartile range) where appropriate. A *p*-value of <0.05 indicates a significant difference. ^a^: Student *t* test; ^b^: Mann–Whitney U test. Abbreviations: ICP: intrahepatic cholestasis of pregnancy; EFW: estimated fetal weight; MVP: maximum vertical pocket; UA: umbilical artery; PI: pulsatility index; S/D: systolic/diastolic ratio.

**Table 3:** Maternal serum afamin levels and biochemical parameters in women with intrahepatic cholestasis of pregnancy and controls.

Variables	ICP (*n* = 40)	Control (*n* = 40)	*p*
Afamin (ng/mL)	6.18 ± 4.24	3.98 ± 1.95	0.004 ^a^
Fasting bile acids (µmol/L)	34.93 ± 29.27	4.49 ± 1.65	<0.001 ^a^
ALT (U/L)	122 (187)	12 (5)	<0.001 ^b^
AST (U/L)	76 (99)	16 (6)	<0.001 ^b^
GGT (U/L)	16 (13)	—	NA
ALP (U/L)	164 (53)	—	NA
Total bilirubin (mg/dL)	0.54 ± 0.27	—	NA
Direct bilirubin (mg/dL)	0.31 ± 0.19	—	NA
Albumin (g/dL)	35.54 ± 2.73	37.01 ± 3.16	0.029 ^a^
PT	8.41 ± 0.55	8.43 ± 0.48	0.838 ^a^
aPTT	27.15 ± 2.66	26.32 ± 2.49	0.153 ^a^
INR	0.89 ± 0.05	0.87 ± 0.05	0.187 ^a^
Fibrinogen (mg/dL)	596.25 ± 108.33	428.03 ± 64.61	<0.001 ^a^

Data are expressed as mean ± SD. A *p*-value of <0.05 indicates a significant difference. ^a^: Student *t* test; ^b^: Mann–Whitney U test; NA: not applicable. Abbreviations: ICP: intrahepatic cholestasis of pregnancy; ng/mL: nanograms per milliliter; µmol/L: micromoles per liter; U/L: units per liter; ALT: alanine aminotransferase; AST: aspartate aminotransferase; GGT: gamma-glutamyl transferase; ALP: alkaline phosphatase; mg/dL: milligrams per deciliter; g/dL: grams per deciliter; PT: prothrombin time; aPTT: activated partial thromboplastin time; INR: international normalized ratio.

**Table 4:** Obstetric and neonatal outcomes in women with intrahepatic cholestasis of pregnancy and controls.

Variables	ICP (*n* = 40)	Control (*n* = 40)	*p*
Gestational age at delivery (weeks)	37 (1)	39 (2)	<0.001 ^a^
Preterm delivery (<37 weeks)	10 (25%)	2 (5%)	0.025 ^b^
Cesarean delivery	21 (52.5%)	21 (52.5%)	1 ^c^
Birth weight (gram)	2858 ± 527	3305 ± 456	<0.001 ^d^
Umbilical cord arterial pH	7.33 (0.11)	7.35 (0.08)	0.042 ^a^
1-min Apgar score	9 (0)	9 (1)	0.050 ^a^
5-min Apgar score	10 (0)	10 (1)	0.044 ^a^
Fetal distress	6 (15%)	3 (7.5%)	0.481 ^b^
Antenatal corticosteroid administration	14 (35%)	0 (0%)	<0.001 ^b^
NICU admission	5 (12.5%)	2 (5%)	0.432 ^b^
TTN	5 (12.5%)	2 (5%)	0.432 ^b^
CPAP	4 (10%)	2 (5%)	0.675 ^b^
RDS	0 (0%)	0 (0%)	NA
Mechanical ventilation	0 (0%)	0 (0%)	NA
Neonatal hypoglycemia	0 (0%)	0 (0%)	NA
Neonatal sepsis	0 (0%)	0 (0%)	NA
Need for phototherapy	1 (2.5%)	0 (0%)	1 ^b^
Perinatal mortality	0 (0%)	0 (0%)	NA
CAPO	15 (37.5%)	5 (12.5%)	0.010 ^c^

Data are expressed as *n* (%), mean ± SD, or median (interquartile range) where appropriate. A *p*-value of <0.05 indicates a significant difference. ^a^: Mann–Whitney U test; ^b^: Fisher’s exact test; ^c^: Pearson chi-square test; ^d^: Student *t*-test. Abbreviations: ICP: intrahepatic cholestasis of pregnancy; NICU: neonatal intensive care unit; TTN: transient tachypnea of the newborn; CPAP: continuous positive airway pressure; RDS: respiratory distress syndrome; CAPO: composite adverse perinatal outcome.

**Table 5:** Comparison of maternal, biochemical, and perinatal outcomes according to fasting bile acid levels in women with intrahepatic cholestasis of pregnancy.

Variables	<40 µmol/L (*n* = 27)	≥40 µmol/L (*n* = 13)	*p*
GA at blood sampling (weeks)	33 (5)	32 (4)	0.360 ^a^
Afamin (ng/mL)	5.71 ± 4.24	7.16 ± 4.25	0.318 ^b^
Fasting bile acids (µmol/L)	18.81 ± 6.42	68.40 ± 29.96	<0.001 ^b^
ALT (U/L)	87 (136)	225 (233)	0.031 ^a^
AST (U/L)	64 (69)	98 (134)	0.073 ^a^
GGT (U/L)	15 (23)	17 (10)	0.549 ^a^
ALP (U/L)	153 (40)	185 (58)	0.083 ^a^
Total bilirubin (mg/dL)	0.49 ± 0.25	0.64 ± 0.28	0.111 ^b^
Direct bilirubin (mg/dL)	0.29 ± 0.17	0.38 ± 0.20	0.151 ^b^
Albumin (g/dL)	35.58 ± 2.71	35.46 ± 2.87	0.901 ^b^
Pre-delivery ALT (U/L)	49 (84)	72 (81)	0.252 ^a^
Pre-delivery AST (U/L)	35 (29)	33 (24)	0.932 ^a^
ALT at 1-week postpartum follow-up (U/L)	25 (29)	24 (31)	0.977 ^a^
AST at 1-week postpartum follow-up (U/L)	22 (14)	14 (26)	0.608 ^a^
Ursodeoxycholic acid dose (250 mg tablets/day)	3 (1)	4 (1)	0.006 ^a^
EFW percentile	44.2 ± 19.5	47.2 ± 24.1	0.679 ^b^
UA PI	0.93 ± 0.21	1.03 ± 0.19	0.168 ^b^
Time from blood sampling to delivery (weeks)	4 (5)	3.5 (3)	0.887 ^a^
Birth weight (gram)	2948 ± 460	2672 ± 624	0.122 ^b^
NICU admission	3 (11.1%)	2 (15.4%)	1 ^d^
NICU length of stay (days)	1.7 ± 7.7	6.8 ± 21.1	0.271 ^b^
CAPO	9 (33.3%)	6 (46.2%)	0.433 ^c^

Data are expressed as *n* (%), mean ± SD, or median (interquartile range) where appropriate. A *p*-value of <0.05 indicates a significant difference. ^a^: Mann–Whitney U test; ^b^: Student *t* test; ^c^: Pearson chi-square test; ^d^: Fisher’s exact test. Abbreviations: GA: gestational age; ng/mL: nanograms per milliliter; µmol/L: micromoles per liter; U/L: units per liter; ALT: alanine aminotransferase; AST: aspartate aminotransferase; GGT: gamma-glutamyl transferase; ALP: alkaline phosphatase; mg/dL: milligrams per deciliter; g/dL: grams per deciliter; EFW: estimated fetal weight; UA: umbilical artery; PI: pulsatility index; NICU: neonatal intensive care unit; CAPO: composite adverse perinatal outcome.

**Table 6:** Correlation analysis between maternal serum afamin levels and clinical, biochemical, and perinatal parameters.

Variables	r	*p*-Value
Maternal Parameters		
Maternal age (years)	0.008	0.959
GA at blood sampling (weeks)	−0.140	0.389
Pre-gestational BMI (kg/m^2^)	0.092	0.573
Biochemical Parameters		
Fasting bile acids (µmol/L)	0.372	0.018
ALT (U/L)	0.127	0.435
AST (U/L)	0.192	0.235
Total bilirubin (mg/dL)	0.166	0.306
Albumin (g/dL)	−0.054	0.740
Fetal/Ultrasonographic Parameters		
EFW percentile	−0.072	0.661
Umbilical artery PI	−0.121	0.455
Neonatal Parameters		
GA at delivery (weeks)	−0.201	0.214
Birth weight (g)	−0.197	0.223
5-min Apgar score	−0.147	0.366
NICU admission	0.004	0.981

A *p*-value of <0.05 indicates a significant difference. Abbreviations: GA: gestational age; BMI: body mass index; µmol/L: micromoles per liter; U/L: units per liter; ALT: alanine aminotransferase; AST: aspartate aminotransferase; mg/dL: milligrams per deciliter; g/dL: grams per deciliter; EFW: estimated fetal weight; PI: pulsatility index; NICU: neonatal intensive care unit.

**Table 7:** ROC Analysis of Maternal Serum Afamin for the Prediction of Intrahepatic Cholestasis of Pregnancy.

	LR+	LR−	Cut-off *	Sensitivity	Specificity	AUC	95% CI	*p*-Value
Afamin	1.69	0.67	>4.93	55%	67.5%	0.634	0.51–0.76	0.039

* Cut-off values were found according to the Youden index. Abbreviations: ROC: receiver operating characteristic; AUC: area under the curve; CI: confidence interval; LR+: positive likelihood ratio; LR−: negative likelihood ratio.

**Table 8:** Univariable and multivariable logistic regression analysis for the prediction of intrahepatic cholestasis of pregnancy.

Variables	Univariable OR	95% CI	*p*-Value	Multivariable * aOR	95% CI	*p*-Value
Afamin	1.254	1.059–1.485	0.009	1.260	1.059–1.500	0.009
GA at blood sampling (weeks)	0.973	0.848–1.116	0.698	1.001	0.861–1.165	0.988
Maternal age (years)	0.991	0.910–1.078	0.829	0.988	0.898–1.087	0.800
Pre-gestational BMI (kg/m^2^)	1.015	0.917–1.124	0.778	1.021	0.911–1.145	0.723
Gestational weight gain (kg)	0.942	0.856–1.037	0.226	0.936	0.845–1.038	0.212

* Multivariable analysis was performed including afamin and all variables evaluated in the univariable analysis. Abbreviations: GA: gestational age; BMI: body mass index; OR: odds ratio; aOR: adjusted odds ratio; CI: confidence interval; kg: kilogram; m: meter.

## Data Availability

The datasets generated and analyzed during the current study are not publicly available due to institutional data protection policies, but are available from the corresponding author on reasonable request.
